# User satisfaction with public oral health services in the Brazilian Unified Health System

**DOI:** 10.1186/s12903-019-0803-8

**Published:** 2019-06-25

**Authors:** Leonardo de Paula Amorim, Maria Inês Barreiros Senna, Gizelton Pereira Alencar, Lorrany Gabriela Rodrigues, Janice Simpson de Paula, Raquel Conceição Ferreira

**Affiliations:** 1 School of Dentistry, Federal University of Minas Gerais, Belo Horizonte, Brazil; 2 Department of Dental Clinic, Pathology and Surgery, School of Dentistry, Universidade Federal de Minas Gerais, Belo Horizonte, Brazil; 3 Department of Epidemiology, Faculty of Public Health, University of São Paulo, São Paulo, Brazil; 4 School of Dentistry, Federal University of Minas Gerais, Belo Horizonte, Brazil; 5 Department of Community and Preventive Dentistry, School of Dentistry, Federal University of Minas Gerais, Belo Horizonte, Brazil

**Keywords:** Patient satisfaction, Oral health, Primary health care, Health services research, Family health, Health evaluation, Dental health services

## Abstract

**Background:**

User satisfaction represents a patient-centered measure that should be used to assess the quality of oral health services. This study investigated the differences in user satisfaction with public oral health services according to the sociodemographic user profile and the quality of oral health services in primary health care in Brazil.

**Methods:**

Secondary data from a national program obtained through interviews with users were analyzed. Satisfaction was based on the Swan’ model relating to perceptions regarding the service performance, assessment of overall satisfaction and the intention to avoid the service in the future. The exploratory variables were demographic characteristics of the users and the quality of the primary service from the user’s viewpoint, considering the dimensions: access; receptivity of spontaneous demand; integral health care; bonding, accountability, and coordination of care.

**Results:**

A total of 37,262 users participated, and 65.51% reported satisfaction with the oral health service, that was higher among those > 20 years old and beneficiaries of the Family Grant Program and lower among users with a higher level of schooling and those who reported being employed. Users who rated oral health service positively were more satisfied.

**Conclusions:**

Socioeconomically disadvantaged user was more satisfied with oral health services and the satisfaction increased with age. The improvement in the quality of oral health services in primary care can result in greater satisfaction.

## Background

Public oral health services in Brazil are mainly provided by oral health teams whose members are the dentist and an oral health assistant and/or an oral health technician. They are part of the healthcare teams of the Family Health Strategy at primary health care units. Since the launch of the National Oral Health Policy [1] in 2004, the number of oral health increased from 20.5% in 2003 [2] to 52.1% in 2018 [3] indicating the large expansion of the public oral health services in Brazil .

The National Oral Health Policy proposed a regular protocol to evaluate the quality of the services and care practices [1]. The implementation of the evaluation process in primary health care in Brazil was consolidated with the National Program for Improving Access to and the Quality of Primary Care (PMAQ-AB) [4], which is nationally standardized by the policies of the National Primary Care Policy [5] and the National Oral Health Policy [1].

The evaluation of services in PMAQ-AB was based on the model proposed by Donabedian, as a conceptual reference that considers the components of structure, process and result. Developed in the 1960s, the model is widely used and is a key reference in the analysis of health services and care practices [6]. The program adopts a set of strategies for the qualification, monitoring and evaluation of health teams linked to a financial incentive to municipalities that meet the access and quality standards and involves several rounds of self- and external assessment. The accreditation process includes the assessment of performance indicators, interviews with health professionals and users as well as facilities assessments in the health units. The quality of oral health services from the perspective of users is rated based on the following dimensions: access; receptivity of spontaneous demand; integral health care; bonding, accountability, coordination of care; and satisfaction with services. The PMAQ-AB was organized in cycles and to date, two cycles have taken place in 2011/2012, 2013/2014. The second cycle of the PMAQ-AB is the most current with consolidated results, and there was a considerable increase in the membership of teams and cities, in addition to the improvement in the reliability of the evaluation instrument. It is therefore important to give continuity to such investigations, including data from the third cycle of PMAQ-AB, which is currently underway [4].

The users’ responses are recognized as indicators of the quality of health services [7, 8]. Indeed, satisfaction is considered a healthcare outcome measure [9, 10]. The evaluation of user satisfaction with health services strengthens the participation of community members by recognizing their civil rights and involving them in the responsibility for the co-production of the health care, which can help guide planning and decision-making processes concerning health management [11].

Recognizing the importance of users in the context of a national program of evaluation of public service quality, this study investigated the differences in user satisfaction with oral health services according to the sociodemographic user profile and the quality of oral health services in primary health care in Brazil.

## Methods

### Study design, population, and sample

An analytical study was conducted using public secondary data from the external evaluation of the second cycle of the PMAQ-AB, which occurred in 2013 and 2014 with the participation of 5072 Brazilian municipalities and 19,946 oral health teams [12]. This study was approved by the Research and Ethics Committee from the Federal University of Minas Gerais (CAEE no. 76981917.4.0000.5149). The public database does not have the participant’s identification to guaranteeing confidentiality.

The questionnaire for the evaluation was constructed and validated by specialists, including technicians and external consultants from the Ministry of Health of Brazil, representatives of states and municipalities, as well as instances of social control. This process was part of project denominated Evaluation for the Improvement of Quality of the Family Health Strategy implemented in 2005. The items of the questionnaire were based on quality standards established according to the norms, protocols, principles, and guidelines to actions and practices organization in primary care [12]. Data were obtained through interviews with four health service users at each primary care unit who did not have a medical, dental or nursing appointment on the day of the interview. This study included data from participants who used oral health services at primary care units. A team of evaluators without a link to the service which had previously undergone training at education and research institutions conducted the interviews.

The methodology used by the PMAQ-AB for the evaluation of the oral health teams, including voluntary adherence to the program, may have resulted in selection bias since the teams that joined might be those with a better structure and work process. Selection bias is also a possibility about the users interviewed, as individuals with no access to health services were not included, which could have led to an underestimation of negative ratings. Thus, satisfaction and the quality of oral health services may be overestimated.

### Assessment of the satisfaction with oral health service

User satisfaction with the oral health service was analyzed based on the four-stage model proposed by Swan, which is defined by the constructs: perception of service performance; confirmation of expectations regarding performance and perception of fair treatment; overall satisfaction; and the intention to revisit or avoid the service in the future [13]. The satisfaction variable was composed of four aspects: i) evaluation of the care received from oral health teams (very good, good, fair, poor or very poor); ii) user satisfaction with the service offered by the dentist (scale of 0 to 10); iii) user satisfaction with the service offered by the oral health assistant/oral health technician (scale of 0 to 10) and iv) the user’s desire to change the dentist or primary care unit if given the option (yes, no). Users who had assessed the received oral health care positively (good or very good), who assigned nine or ten points for satisfaction with the care received from the dentist and oral health assistant/oral health technician and those reported that they would not change the dentist or primary care unit were considered satisfied. Users with any other combination of answers were considered unsatisfied.

### Exploratory variables

#### Sociodemographic user profile

The sociodemographic user profile variables were gender (male; female), age group, self-reported skin color/race, years of schooling, currently formally or informally employed (yes; no) and beneficiary of the Brazilian Family Grant Program (yes; no). Age group in years was categorized based on the lifecycle: adolescent (16 to 19 years), young adult (20 to 39 years), adult (40 to 59 years) and elder (60 years and over). Self-reported skin color/race was evaluated as white, black, yellow and brown or indigenous, according to the classification of the Brazilian Institute of Geography and Statistics [14]. Considering the stages of basic education in Brazil, schooling was categorized as illiterate or functionally illiterate (including the original non-literate categories and the ability to read and write), one to eight years of study, nine to 11 years of study and 12 or more years of study.

#### Evaluation of quality of oral health services regarding primary care attributes from the perspective of users

The users rated the quality of the health services addressing primary care attributes according to the proposal of the PMAQ-AB: access, receptivity of spontaneous demand for care, integral health care, ties, accountability, and coordination of care. The selected variables were described in Table 1.

**Table 1:** Variables to the evaluation of the quality of oral health services by users

Access to oral health services	
1 Does the team announce office hours of the unit?	No; Yes
2 Do the office hours of the dental service meet your needs?	No; Yes
Receptivity of spontaneous demand	
3 When do you seek dental care without an appointment, are listened to?	No; Yes, sometimes: Yes, always
Integral health care	
4 Is the dental office a reserved place (is there privacy)?	No; Yes
5 During consultations, how often are you advised by oral health professionals regarding oral health care?	Always; Most of the time; Almost never; Never
6 During consultations, does the professional make notes in your medical record or card?	No; Yes, sometimes; Yes, always
Bonding, accountability, and coordination of care	
7 During consultations with oral health professionals, do you think the query time is enough?	No; Yes, sometimes; Yes, always
8 At this health unit, are you treated by the same dentist?	Never; Almost never; Most of time; Always;
9 When you stop treatment for any reason or do not appear for a dental appointment, do the professionals make contact to ask you what happened and resume the care?	Never abandoned or missed; No; Yes, sometimes; Yes, always

Source: External evaluation questionnaire of the second cycle of the PMAQ-AB, module III, 2013

### Statistical analysis

The data were submitted to descriptive analysis. Associations between user satisfaction and variables related to the sociodemographic user profile and the quality of oral health services (access; receptivity of spontaneous demand; integral health care; bonding, accountability, and coordination of care) were investigated through multiple logistic regression analyses performed with the software Stata 14.0. For the evaluation of the goodness-of-fit of the model, residuals analysis based on the standardized Pearson residue and deviance was performed according to Hosmer, Lemeshow, and Sturdivant (2013) [15]. Three users were identified as having very high deviance residual and were excluded from the final model. After the residual analysis, Hosmer Lemeshow’s modified goodness-of-fit test for large samples (number of groups = number of individuals/2) was used [16].

## Results

A total of 114,615 users, among whom 37,262 (32.51%) used oral health services, were included in the present analysis. Most users were covered by family health unit with an oral health team (95.34%). More than 70% of users gave positive responses to questions on satisfaction (Table 2). Considering the set of selected items, 65.51% (*n* = 24,412) of the users were satisfied.

**Table 2:** Distribution of users concerning satisfaction with oral health services

Variables	n	%
Satisfaction with care and with professionals on the oral health team		
Evaluation of the care received by the oral health team		
Very poor, poor or fair	4.352	11.74
Good or very good	32.738	88.27
User satisfaction with the service offered by the dentist		
0 to 8	8.721	23.41
9 or 10	28.541	76.59
User satisfaction with the service offered by the oral health assistance/technician		
0 to 8	10.445	28.03
9 or 10	26.817	71.97
User’s desire to change the dentist or primary care unit if given the option		
No	5.410	14.52
Yes	31.852	85.48

Table 3 shows the distribution of the satisfaction with oral health services according to the exploratory variables. A low satisfaction was found among users who reported that the dental agenda is inadequate to user schedule, those who reported never or nearly never receiving oral health advice, those who reported that the dentist did not record the oral findings in the medical records, those who reported that the time allotted for the dental appointment was insufficient and those who reported never being seen by the same dentist.

**Table 3:** Proportion of users according to sociodemographic user profile and quality of oral health care

Variables	Total sample n (%)	Satisfaction with oral health services n (%)
Sociodemographic user profile		
Sex		
Male	6.514 (17.48)	4.280 (65.70)
Female	30.748 (82.52)	20.132 (65.47)
Age group (years of age)		
Adolescent (16–9)	1.821 (4.89)	1.006 (55.24)
Young adult (20–39)	18.981 (50.94)	11.928 (62.84)
Adult (40–59)	11.733 (31.49)	8.012 (68.29)
Elder (60 or more)	4.727 (12.69)	3.466 (73.32)
Skin color or race		
White	12.797 (34.92)	8.480 (66.27)
Black	4.723 (12.89)	3.069 (64.98)
Yellow	1.151 (3.14)	708 (61.51)
Brown or indigenous	17.978 (49.05)	11.751 (65.36)
Schooling		
Illiterate or functionally illiterate	3.509 (9.43)	2.610 (74.38)
1 to 8 years of study	16.974 (45.60)	11.531 (67.93)
9 to 11 years of study	14.057 (37.76)	8.607 (61.23)
12 or more years of study	2.683 (7.21)	1.635 (60.94)
Beneficiary of the Family Grant Program		
No	22.277 (59.95)	14.509 (65.13)
Yes	14.885 (40.05)	9.836 (66.08)
Formally or informally employed		
No	23.625 (63.40)	15.712 (66.51)
Yes	13.637 (36.60)	8.700 (63.80)
Access to oral health services		
Oral health team announces office hours		
Does not announce	6.726 (18.05)	3.625 (53.90)
Announces	30.536 (81.95)	20.787 (68.07)
Dental agenda is adequate to user schedule		
Does not meet	4.557 (12.59)	1.539 (33.77)
Meets	31.652 (87.41)	22.380 (70.71)
Receptivity of spontaneous demand		
Listened to when looking for care without an appointment		
No	15.556 (41.75)	8.821 (56.70)
Yes, sometimes	5.952 (15.97)	3.489 (58.62)
Yes, always	15.754 (42.28)	12.102 (76.82)
Integral health care		
Privacy/place reserved		
No	1.882 (5.08)	757 (40.22)
Yes	35.167 (94.92)	23.559 (66.99)
Receive advice on oral health		
Never	1.952 (5.29)	530 (27.15)
Almost never	1.290 (3.49)	336 (26.05)
Most of the time	5.436 (14.73)	2.917 (53.66)
Always	28.238 (76.49)	20.495 (72.58)
Dentist makes notes on dental records		
No	1.183 (3.33)	427 (36.09)
Yes, sometimes	1.419 (4.00)	624 (43.97)
Yes, always	32.912 (92.67)	22.467 (68.26)
Bonding, accountability, and coordination of care		
Query time enough		
No	2.118 (5.73)	366 (17.28)
Yes, sometimes	3.415 (9.25)	1.348 (39.47)
Yes, always	31.403 (85.02)	22.567 (71.86)
Serviced by the same dentist		
Never	586 (1.60)	183 (31.23)
Almost never	2.023 (5.54)	848 (41.92)
Most of the time	6.580 (18.01)	3.904 (59.33)
Always	27.340 (74.84)	19.176 (70.14)
Contacted when having missed an appointment		
No	9.246 (25.19)	4.337 (46.91)
Yes, sometimes	1.767 (4.81)	1.196 (67.69)
Yes, always	5.397 (14.71)	4.393 (81.40)
Never abandoned or missed	20.289 (55.28)	14.208 (70.03)

The final regression model shows that the chance of satisfaction with oral health services was higher among users over 20 years of age and beneficiaries of the Family Allowance Program, whereas satisfaction was lower among users with a higher level of schooling and who reported being employed. Users who perceived better quality regarding oral health services considering the attributes of primary care reported greater satisfaction (Table 4).

**Table 4:** Factors associated with satisfaction with oral health services in multivariate logistics analysis

Variables	Crude OR (95%IC)	Adjusted OR (95%IC)
Sociodemographic user profile		
Sex		
Male	1	
Female	0.99 (0.94–1.05)	
Age group (years)		
Adolescent (16–9)	1	1
Young adult (20–39)	**1.37 (1.24–1.51)**	**1.24 (1.10–1.40)**
Adult (40–59)	**1.74 (1.58–1.93)**	**1.37 (1.21–1.55)**
Elder (60 or more)	**2.23 (1.99–2.49)**	**1.57 (1.36–1.81)**
Skin color/race		
White	1	1
Black	0.94 (0.88–1,01)	0.98 (0.90–1.06)
Yellow	**0.81 (0.72–0,92)**	0.92 (0.80–1.07)
Brown/mestizo or indigenous	0.96 (0.92–1,01)	**1.06 (1.00–1.12)**
Schooling		
Illiterate or functionally illiterate	1	1
1 to 8 years of study	**0.73 (0.67–0.79)**	**0.75 (0.68–0.83)**
9 to 11 years of study	**0.54 (0.50–0.59)**	**0.61 (0.54–0.68)**
12 or more years of study	**0.54 (0.48–0.60)**	**0.58 (0.50–0.67)**
Formally or informally employed		
No	1	1
Yes	**0.89 (0.85–0.93)**	**0.91 (0.86–0.96)**
Beneficiary of the Family Grant Program		
No	1	1
Yes	**1.04 (1.00–1.09)**	**1.13 (1.07–1.20)**
Access to oral health services		
Oral health team announces office hours		
Does not announce	1	1
Announces	**1.83 (1.73–1.93)**	**1.18 (1.10-.,27)**
Dental agenda is adequate to user schedule		
Does not meet	1	1
Meets	**4.74 (4.44–5.07)**	**2.22 (2.05–2.41)**
Receptivity of spontaneous demand		
Listened to when looking for care without an appointment		
No	1	1
Yes, sometimes	**1.08 (1.02–1.15)**	1,05 (0,97-1,13)
Yes, always	**2.53 (2.41–2.66)**	**1.62 (1.52–1.71)**
Integral health care		
Privacy/place reserved		
No	1	1
Yes	**3.02 (2.75–3.32)**	**1.79 (1.59–2.02)**
Receive advice on oral health		
Never	1	1
Almost never	0.95 (0.81–1.11)	1.05 (0.86–1.28)
Most of the time	**3.12 (2.79–3.50)**	**2.19 (1.90–2.53)**
Always	**7.14 (6.44–7.92)**	**3.40 (2.98–3.88)**
Dentist makes notes on dental records		
No	1	1
Yes, sometimes	**1.40 (1.19–1.64)**	**1.27 (1.04–1.54)**
Yes, always	**3.83 (3.39–4.32)**	**1.54 (1.32–1.80)**
Bonding, accountability, and coordination of care		
Query time enough		
No	1	1
Yes, sometimes	**3.15 (2.76–3.59)**	**2.09 (1.79–2.44)**
Yes, always	**12.33 (10.98–13.84)**	**4.81 (4.19–5.51)**
Serviced by the same dentist		
Never	1	1
Almost never	**1.60 (1.31–1.94)**	1.12 (0.87–1.45)
Most of the time	**3.23 (2.69–3.87)**	**1.72 (1.36–2.18)**
Always	**5.20 (4.36–6.21)**	**2.24 (1.78–2.82)**
Contacted when having missed appointment		
No	1	1
Yes, sometimes	**2.37 (2.13–2.64)**	**1.81 (1.59–2.05)**
Yes, always	**4.96 (4.58–5.37)**	**2.52 (2.30–2.76)**
Never abandoned or missed	**2.65 (2.52–2.78)**	**1.88 (1.77–2.00)**

The bolded refect statistically significant findings

Hosmer and Lemeshow test result, modified for large samples = 0.2182

## Discussion

Most users reported satisfaction with oral health services and the satisfaction was higher among those who evaluated the oral health services about the attributes of primary care positively. Age and socioeconomic status were associated with satisfaction with oral health services.

The satisfaction with oral health services was higher among beneficiaries of the Family Grant Program. It is a national cash transfer program that stipulates conditions for beneficiaries regarding the use of health services and education. Families receiving this benefit must keep their school-age children enrolled as well as adopt basic health measures, including immunization and prenatal monitoring [17]. The program integrates a political agenda of social protection and reduction of inequities in health and favored access to health services of a population historically socially excluded, with positive effects in reducing child mortality [18], health conditions and well-being [19]. This association between unfavorable social conditions and a higher frequency of satisfaction was also identified among those with a lower level of education and unemployed. These findings indicate that the social inclusion of these groups, through access to health services, in addition to promoting equity, prompt satisfaction with the oral health service.

The satisfaction increased with aging. This finding is in agreement with data reported in a previous study conducted in Brazil, in which the youngest participants reported the least satisfaction with oral health services [20]. Similar findings were reported in studies conducted in the primary care setting in Saudi Arabia [21] as well as at hospital services in Sweden [22] and the United States [23]. A dose-response gradient was also found concerning schooling, as a lower level of schooling favored greater levels of satisfaction. As pointed out in a study evaluating user satisfaction with primary care in a small Brazilian city, a low level of education can exert an influence on the perception of health problems and lead to a less critical value judgment regarding the care received [24].

Users who gave better ratings of the oral health teams about the quality attributes of primary care reported greater satisfaction with oral health services. Concerning the access dimension, a higher satisfaction was found among those who reported that the dental agenda is adequate to user schedule and that the oral health team announces office hours. A study evaluating the perception of users regarding the organization of Brazilian public dental services pointed out that convenience concerning dental office hours favored satisfaction [25]. In the present investigation, users reported that most of the oral health teams worked in two shifts: morning and afternoon (77.89%) (data not shown). It is possible that those who were unsatisfied and reported that dental agenda is inadequate to user schedule have daily activities that hinder the use of the service. The organization of the agenda including diversified shifts and a night shift could expand access and enhance satisfaction. Indeed, offering evening appointments are considered a relevant indicator in assessing the quality of oral health care [26].

Higher satisfaction was observed among users who were listened when looking for services without an appointment than those that were not. Although receptivity is a guiding aspect of the National Policy on Humanization [27], a narrative review highlighted a shortcoming in the training of dentists and managers regarding the challenges of receptivity in the practice of oral health care [28]. One study pointed out that the humanization of care, as well as listening and guidance processes, were associated with the quality of receptivity at oral health services [29]. Thus, the improvement of the quality of oral health care should not be restricted to the technical training of the team, but also to attempt to develop the listening skill aiming to meet the needs and expectations of users.

Users who reported that dental care was offered in private room, who always or often received guidance on oral health and that the dentist recorded information in the medical reports reported greater satisfaction. The absence of privacy due to sharing the same dental office environment can be a source of embarrassment and feelings of dissatisfaction due to the breach of confidentiality, shame in showing one’s mouth to others or insecurity. This aspect of quality is in line with the recommendation of the National Policy on Humanization in Brazil, which encourages the creation of healthy spaces that respect privacy and encourage the work process [27]. In this sense, there is a need for improvements in the structure of the primary care units and dental facilities. The guidance given to users represents a form of care and attention. The qualification of dialogue has the potential to strengthen ties as well as enhance the role of the user in the health professional-patient relationship. Access to information is a right and fosters empowerment and literacy in health [30], the importance of which is evidenced, as it encourages user involvement in the decision-making process [31, 32] and increases levels of satisfaction with oral health services [33]. A German study showed that better explanations regarding oral health care were associated with more satisfied users [9].

Similarly, users were more unsatisfied when they did not receive information on how to prevent oral problems in a Brazilian study conducted in a medium-sized town [33]. The perception that the dentist records information from the session in the dental-medical records was associated with greater satisfaction. Health records encourage the coordination of care in a multidisciplinary perspective based on the patient-centered model [34] and are an indicator of the quality of care [35].

Regarding the bonding, accountability, and coordination of care domains, positive assessments were associated with greater satisfaction with oral health service. A dose-response gradient was found for these three variables, with a greater chance of user satisfaction when the time allotted for the appointment was considered “always sufficient”, when the patient was “always” treated by the same dentist and contact with the patient was “always” made when he/she missed a dental appointment. A Canadian study showed that a close professional-patient relationship based on the sharing of power favors a patient-centered rather than disease-centered approach and proposed an user-centered dental care model. Meeting care expectations increases the satisfaction of both the patient and professional and reduces tension resulting from patient frustration. The proposed model enables a contextualization of the problem, the consolidation of ties and continual care [36]. In a study evaluating primary care at a government agency (Veterans Health Administration), higher levels of patient satisfaction were found when primary care attributes, such as the coordination of care and shared decision-making, were considered adequate [37].

The present national study in the field of health evaluation has strong points and limitations. While a sample with a large number of participants ensures representativeness, associations between variables tend to be significant. However, the residual analysis and the goodness-of-fit test revealed a suitable model. Although the questionnaires used for data collection in the PMAQ were validated by experts, the evaluation of reliability was not performed. On the other hand, the organization of the program in cycles allows continuous improvement of the issues.

## Conclusions

In this national study, user satisfaction with public oral health services was high and the attributes of the primary care that received positive evaluations by users favored satisfaction with the oral health service. The socioeconomically disadvantaged user was more satisfied with oral health services and the satisfaction increased with age. These results suggest that the unfavorable social conditions can be associated with different user expectations about oral health services which in turn could affect the satisfaction level and the evaluation of the quality of services.

## Data Availability

The datasets analyzed during the current study are available in the Portal of the Department of Primary Care repository, http://dab.saude.gov.br/portaldab/ape_pmaq.php?conteudo=2_ciclo [12].

## Abbreviations

OR: Odds ratio
PMAQ-AB: National Program for Improving Access to and the Quality of Primary Care
