# Correction to: User satisfaction with public oral health services in the Brazilian unified health system

**DOI:** 10.1186/s12903-019-0911-5

**Published:** 2019-10-15

**Authors:** Leonardo de Paula Amorim, Maria Inês Barreiros Senna, Gizelton Pereira Alencar, Lorrany Gabriela Rodrigues, Janice Simpson de Paula, Raquel Conceição Ferreira

**Affiliations:** 1 School of Dentistry, Federal University of Minas Gerais, Belo Horizonte, Brazil; 2 Department of Dental Clinic, Pathology and Surgery, School of Dentistry, Universidade Federal de Minas Gerais, Belo Horizonte, Brazil; 3 Department of Epidemiology, Faculty of Public Health, University of São Paulo, São Paulo, Brazil; 4 School of Dentistry, Federal University of Minas Gerais, Belo Horizonte, Brazil; 5 Department of Community and Preventive Dentistry, School of Dentistry, Federal University of Minas Gerais, Belo Horizonte, Brazil

**Correction to: BMC Oral Health.**

**https://doi.org/10.1186/s12903-019-0803-8**

Following publication of the original article [1], the authors have reported that there is an error in Table 2 - Distribution of users concerning satisfaction with oral health services: the categories ‘No’ and ‘Yes’ should swap places.

Hence:

‘Yes’ would be in the same line with the values *n* = 5.410 and % = 14.52;

‘No’ would be in the same line with the values *n* = 31.852 and % = 85.48.
